# Airborne PM_2.5_ Chemical Components and Low Birth Weight in the Northeastern and Mid-Atlantic Regions of the United States

**DOI:** 10.1289/ehp.1104763

**Published:** 2012-09-20

**Authors:** Keita Ebisu, Michelle L. Bell

**Affiliations:** School of Forestry and Environmental Studies, Yale University, New Haven, Connecticut, USA

**Keywords:** air pollution, environmental health, epidemiology, low birth weight

## Abstract

Background: Previous studies on air pollutants and birth outcomes have reported inconsistent results. Chemical components of particulate matter ≤ 2.5 µm (PM_2.5_) composition are spatially -heterogeneous, which might contribute to discrepancies across PM_2.5_ studies.

Objectives: We explored whether birth weight at term is affected by PM_2.5_, PM_10_ (PM ≤ 10 µm), and gaseous pollutants.

Methods: We calculated exposures during gestation and each trimester for PM_2.5_ chemical components, PM_10_, PM_2.5_, carbon monoxide, nitrogen dioxide, ozone, and sulfur dioxide for births in 2000–2007 for states in the northeastern and mid-Atlantic United States. Associations between exposures and risk of low birth weight (LBW) were adjusted by family and individual characteristics and region. Interaction terms were used to investigate whether risk differs by race or sex.

Results: Several PM_2.5_ chemical components were associated with LBW. Risk increased 4.9% (95% CI: 3.4, 6.5%), 4.7% (3.2, 6.2%), 5.7% (2.7, 8.8%), and 5.0% (3.1, 7.0%) per interquartile range increase of PM_2.5_ aluminum, elemental carbon, nickel, and titanium, respectively. Other PM_2.5_ chemical components and gaseous pollutants showed associations, but were not statistically significant in multipollutant models. The trimester associated with the highest relative risk differed among pollutants. Effect estimates for PM_2.5_ elemental carbon and nickel were higher for infants of white mothers than for those of African-American mothers, and for males than females.

Conclusions: Most exposure levels in our study area were in compliance with U.S. Environmental Protection Agency air pollution standards; however, we identified associations between PM_2.5_ components and LBW. Findings suggest that some PM_2.5_ components may be more harmful than others, and that some groups may be particularly susceptible.

Adverse birth outcomes, such as low birth weight (LBW), increase risk of mortality and morbidity, including cardiovascular-related events, during childhood (Kannan et al. 2006). In the last decade, numerous studies have reported associations between levels of ambient air pollutants and adverse birth outcomes, although results are not consistent regarding the relevance of specific pollutants or the trimester of exposure. Associations between particulate matter (PM) and pregnancy outcomes differ by study, although many findings do suggest an association. Exposure to PM ≤ 10 µm (PM_10_) and 2.5 µm (PM_2.5_) in aerodynamic diameter during gestation has been associated with LBW in some studies (Huynh et al. 2006; Morello-Frosch et al. 2010; Seo et al. 2010), but not others (Madsen et al. 2010; Seo et al. 2010). More studies have been conducted for gaseous pollutants, although results have also been inconsistent, such as for nitrogen dioxide (NO_2_) (Maroziene and Grazuleviciene 2002; Morello-Frosch et al. 2010), sulfur dioxide (SO_2_) (Bobak and Leon 1999; Lin et al. 2004), carbon monoxide (CO) (Liu et al. 2003; Ritz and Yu 1999), and ozone (O_3_) (Morello-Frosch et al. 2010; Salam et al. 2005).

Several literature reviews of pollutant effects on adverse birth outcomes have noted that results are heterogeneous across studies, but have nonetheless concluded that associations between air pollution and adverse pregnancy outcomes are likely causal (Maisonet et al. 2004; Sapkota et al. 2010; Shah and Balkhair 2011). Shah and Balkhair (2011) concluded that exposure to PM_2.5_ is likely associated with LBW, preterm birth, and small-for-gestational-age births. These reviews noted that further studies are necessary to clarify which pollutants are the most harmful and to identify during which periods of pregnancy infants are most vulnerable. Inconsistencies among previous studies might result from differences in study populations or in study design, such as control for confounders, exposure assessment, statistical methods, and sample size. Other possible explanations are variation in the exposure period and collinearity among pollutants (Maisonet et al. 2004). However, a key reason studies on PM and pregnancy outcomes differ is that the chemical composition of particles varies by location (Bell et al. 2007a). Previous works demonstrated regional variation in the chemical structure of PM_2.5_ (Bell et al. 2007a) and in PM_2.5_-associated risk for mortality (Zhou et al. 2011). Several studies have used data on components or sources to investigate whether associations between PM and adverse outcomes are related to chemical composition. In the United States, relative risks of cardiovascular hospitalizations in association with PM_2.5_ total mass are higher in areas with higher PM_2.5_ content of bromine, chromium, nickel, and sodium (Zanobetti et al. 2009). In California, long-term exposures to fossil fuel–related PM_2.5_ (e.g., sulfate) and crustal-related PM_2.5_ (e.g., silicon) are associated with increased mortality (Ostro et al. 2010). In New York City, the effect of coal combustion–related components (e.g., selenium) on cardiovascular-related mortality is higher in summer than in winter, whereas its effect on cardiovascular-related hospital admission is higher in summer than in winter (Ito et al. 2011).

Most studies of PM_2.5_ sources or components have focused on adult hospital admissions or mortality, with only a limited number of studies investigating associations between PM_2.5_ chemical components and birth outcomes. A study conducted in Atlanta, Georgia, reported that PM_2.5_ elemental carbon and water-soluble PM_2.5_ metals, such as copper, were associated with lower birth weight (Darrow et al. 2011). Our previous studies of four counties in Connecticut and Massachusetts found associations between PM_2.5_ components of aluminum, elemental carbon, nickel, silicon, vanadium, and zinc and risk of LBW (Bell et al. 2010).

Given the spatially heterogeneous distribution of PM_2.5_ components (Bell et al. 2007a), there is value in investigating effects of components over a larger spatial area and associations with components such as organic carbon matter (OCM) that have not been considered previously. In the present study, we investigated associations between exposure to PM_10_, PM_2.5_ total mass, PM_2.5_ chemical components, CO, NO_2_, O_3_, and SO_2_ during pregnancy and birth weight for the Northeastern and Mid-Atlantic regions of the United States. In previous work, we investigated associations between ambient air pollution and pregnancy outcomes in Connecticut and Massachusetts, but did not consider some key PM_2.5_ chemical components such as OCM (Bell et al. 2010) or did not consider any PM_2.5_ chemical components at all (Bell et al. 2007b). Compared with our previous studies, the present study covers a much larger study area and a population that is 16 times larger, expands the components considered, and evaluates research questions not considered previously, such as potential confounding by gaseous pollutants. To the best of our knowledge, this is the largest study to date of the effects of PM_2.5_ chemical components on birth weight.

The National Research Council Committees and the Health Effects Institute identified as a critical research need studies on which characteristics of particles are most harmful (Health Effects Institute 2002; National Research Council 2004). Scientific evidence on the health impacts of PM_2.5_ components will inform understanding of which sources are most harmful and will benefit policy making efforts to protect public health from airborne PM_2.5_.

## Methods

*Birth data.* Birth certificate data for Connecticut, Delaware, Maryland, Massachusetts, New Hampshire, New Jersey, New York, Pennsylvania, Rhode Island, Vermont, Virginia, Washington, DC, and West Virginia (USA), from 1 January 2000 through 31 December 2007 were obtained from the National Center for Health Statistics (Atlanta, GA). Data that were provided include county of residence, county of birth, birth order, trimester of first prenatal care, date of last menstrual period (LMP), gestational age, infant’s sex and birth weight, as well as maternal and paternal ages and races, and maternal education, marital status, alcohol consumption, and smoking during pregnancy. Further description of these data is available elsewhere (Bell et al. 2007b).

Births with unspecified county of residence or birth, plural deliveries (e.g., twins), gestational period > 44 weeks, gestational period < 37 weeks (nonterm births), birth weight < 1,000 g or > 5,500 g, different counties of residence and delivery, or impossible gestational age and birth weight combinations were excluded from analysis (Alexander et al. 1996). Births also were excluded if LMP was missing or the estimated birth based on LMP and gestational length was > 30 days from the midday of the birth month reported on the birth certificate.

*Air pollution and weather data.* PM_2.5_ chemical components data were obtained from the U.S. Environmental Protection Agency (EPA) Air Explorer (U.S. EPA 2010a). PM_10_ and PM_2.5_ total mass, CO, NO_2_, O_3_, and SO_2_ data were obtained from the U.S. EPA Air Quality System for 1999–2007 (U.S. EPA 2010b). We included only counties with PM_2.5_ chemical component data because these exposures are our primary focus. PM_10_, PM_2.5_, and PM_2.5_ chemical components were measured every 3–6 days. Gaseous pollutants were measured daily, although O_3_ was measured mainly during the warm season. Some monitors began or ceased observation during the study period. We investigated PM_2.5_ chemical components identified by previous research and literature review to have potential links to health and/or contribute substantially to PM_2.5_ total mass: aluminum, ammonium ion, arsenic, cadmium, calcium, chlorine, elemental carbon, lead, mercury, nickel, nitrate, organic carbon matter, silicon, sodium ion, sulfur, titanium, vanadium, and zinc (Bell et al. 2007a; Franklin et al. 2008; Haynes et al. 2011; Ostro et al. 2007; Zanobetti et al. 2009).

We calculated apparent temperature (AT), a measure that reflects overall temperature discomfort (Kalkstein and Valimont 1986), based on daily temperature and dew point temperature data obtained from the National Climatic Data Center (2010). If weather data were unavailable for a given county, we assigned the AT value for the closest county with weather data.

*Exposure estimation.* For each birth we calculated the average level of each pollutant during gestation and each trimester, and average AT during each trimester. Delivery date was estimated based on self-reported LMP and gestational length, assuming conception 2 weeks after LMP. We defined trimesters as 1–13 weeks, 14–26 weeks, and week 27 to delivery, as in previous studies (Bell et al. 2007b).

Exposures were estimated based on county of residence. Not all counties had data for all pollutants. Measurements from multiple monitors in the same county on the same day were averaged to generate daily pollutant -levels. To avoid biases due to changes in measurement frequency, daily pollutant levels and AT values were combined to estimate weekly exposures, which were then averaged to estimate gestational or trimester exposure. Births for which exposure estimates were unavailable for > 25% of the weeks in any trimester for a given pollutant were excluded from analyses of that pollutant.

*Statistical analysis.* Each birth was cate-gorized as low or normal birth weight using clinically defined LBW (< 2,500 g). Logistic regression was used to estimate associations between LBW and gestational exposure to each pollutant with adjustment for maternal race (African American, Caucasian, other), marital status (married, unmarried), tobacco consumption during pregnancy (yes, no, unknown), alcohol consumption during pregnancy (yes, no, unknown), highest education (< 12 years, 12 years, 13–15 years, > 15 years, unknown), age (< 20, 20–24, 25–29, 30–34, 35–39, ≥ 40 years), infant sex (male, female), gestational length (37–38, 39–40, 41–42, 43–44 weeks), the trimester prenatal care began (1st, 2nd, 3rd, no care, unknown), first in birth order (yes, no, unknown), delivery method (vaginal, cesarean section, unknown), average AT for each trimester, season of birth, and year of birth. In addition we included regional indicators to adjust for local factors such as area-level socioeconomic conditions (Table 1). We conducted sensitivity analyses restricted to first births to assess the influence of multiple births by the same mother on associations (Zhu et al. 1999).

**Table 1:** Characteristics of study births (*n* = 1,207,800).

Characteristic	Mean ± SD or *n* (%)
Birth weight (g)	3385.9 ± 472.8
Low birth weight (< 2,500 g)	34,038 (2.8)
Sex
Male	614,923 (50.9)
Female	592,877 (49.1)
Race
White	789,682 (65.4)
African American	305,798 (25.3)
Other	112,320 (9.3)
Maternal age (years)
< 20	99,017 (8.2)
20 to 24	249,077 (20.6)
25 to 29	323,322 (26.8)
30 to 34	324,221 (26.8)
35 to 39	173,093 (14.3)
≥ 40	39,070 (3.2)
Maternal marital status
Married	720,088 (59.6)
Single	487,712 (40.4)
Maternal education
Less than high school	214,063 (17.7)
High school	327,399 (27.1)
Some college	261,206 (21.6)
College	394,363 (32.7)
Unknown	10,769 (0.9)
Maternal alcohol consumption during pregnancy
Yes	3,785 (0.3)
No	688,991 (57.1)
Unknown	515,024 (42.6)
Maternal tobacco consumption during pregnancy
Yes	94,559 (7.8)
No	1,106,456 (91.6)
Unknown	6,785 (0.6)
Length of gestation (weeks)
37 to 38	341,094 (28.2)
39 to 40	639,772 (53.0)
41 to 42	193,700 (16.0)
43 to 44	33,234 (2.8)
Month prenatal care begin
First 3 months of pregnancy	949,081 (78.6)
4th to 6th months of pregnancy	184,828 (15.3)
7th month of pregnancy or later	44,938 (3.7)
No care	6,822 (0.6)
Unknown	22,131 (1.8)
Birth order
First baby	404,233 (33.5)
Not first baby	795,322 (65.9)
Unknown	8,245 (0.7)
Region
Connecticut	33,246 (2.8)
Delaware	40,412 (3.4)
Massachusetts	51,674 (4.3)
Maryland and DC	111,187 (9.2)
New Hampshire	26,314 (2.2)
New Jersey	146,508 (12.1)
Manhattan area, New York	344,901 (28.6)
New York other than Manhattan area	82,194 (6.8)
Eastern Pennsylvania	203,428 (16.8)
Western Pennsylvania	100,355 (8.3)
Rhode Island	31,108 (2.6)
Virginia	20,399 (1.7)
Vermont	8,653 (0.7)
West Virginia	7,421 (0.6)
Birth year
2000	8,809 (0.7)
2001	35,821 (3.0)
2002	123,951 (10.3)
2003	202,008 (16.7)
2004	180,076 (14.9)
2005	213,465 (17.7)
2006	219,494 (18.2)
2007	224,176 (18.6)
Birth season
Winter	286,495 (23.7)
Spring	291,176 (24.1)
Summer	310,798 (25.7)
Fall	319,331 (26.4)

For pollutants showing statistically significant associations with LBW in single--pollutant models, we conducted two-pollutant models that included pairs of pollutants that were not highly correlated (correlation < 0.5). Similarly, for pollutants associated in single-pollutant model, we assessed effects by trimester using a model with trimesters’ exposures included simultaneously. Because trimester exposures could be correlated, we performed sensitivity analysis with trimester exposures adjusted to be orthogonal using a method we published previously (Bell et al. 2007b). In brief, we predicted exposures of two trimesters using exposure level of a given trimester (reference trimester), calculated their residuals, and put them into models besides exposure of reference trimester. This approach can avoid covariance among trimester exposures. This procedure was repeated using each trimester as the reference trimester, and we have four models for trimester analysis in total (main model and three models as sensitivity -analyses). Further description of this approach is available elsewhere (Bell et al. 2007b).

Additional analyses were conducted for pollutants that showed statistically robust results in two-pollutant models. We included interaction terms between gestational pollutant exposures and sex or race to investigate whether some populations are particularly susceptible, because previous analysis found higher relative risks associated with ambient air pollution in some populations than in others (Bell et al. 2007b). Statistical significance was determined at an alpha level of 0.05 for the entire analyses.

## Results

There were 7,098,417 births in 419 counties in the study area during the study time period (2000–2007). Among them, 2,476,383 (34.9%) infants lived in the 50 counties with monitors for PM_2.5_ chemical components, and 1,385,466 (19.5%) infants in 49 counties had exposure estimates for all pollutants during ≥ 75% of the gestational weeks in all three trimesters. After exclusions (e.g., for preterm birth, plural deliveries), our study population consisted of 1,207,800 infants from 49 counties. This corresponds to 17% of the original data, and some births may have been excluded based on more than one criterion. Many of the counties had only one monitor, but some urban counties had multiple monitors. The average number of monitors per county was 1.08 (range, 1–2) for PM_2.5_ chemical components, and 1.57 (range, 1–9) for PM_10_, PM_2.5_, and gaseous pollutants. The mean (± SD) area of the 49 counties is 540.5 ± 395.3 mi^2^ (median = 528.6 mi^2^), and average population was 511,146 ± 453,846 (median = 433,501). About three-quarters of the monitors were located in urban or suburban areas, and the remainder were located in rural areas. In the 49 counties, the average urbanicity rate based on 2000 U.S. Census values was 81.7% (median 92.1%, minimum 13.8%, maximum 100.0%) (U.S. Census Bureau 2000).

Average male birth weight was 3446.9 ± 478.2 g, with 2.3% LBW. Female birth weight was 3322.7 ± 458.5 g, with 3.4% LBW. About two-thirds of mothers were Caucasian and one-quarter were African American (Table 1). More than 80% of mothers had high school or higher education. From those born in 2000 and 2001, we had fewer study subjects compared with other years because fewer PM_2.5_ chemical component monitors were in -operation during that time.

Births that were excluded because of a lack of monitors but were otherwise eligible were similar to births included in the analysis [see Supplemental Material, Table S1 (http://dx.doi.org/10.1289/ehp.1104763)], but included a higher fraction of white mothers (77.5% vs. 65.4%), a lower fraction of African-American mothers (16.2% vs. 25.3%), and a higher fraction of married mothers (68.1% vs. 59.6%). Births excluded for reasons other than a lack of monitors differed with regard to exclusion criteria (e.g., gestational week, birth weight), but were similar to study births with respect to -mother’s race, age, marital status, and education.

Table 2 shows gestational exposure to pollutants overall, and Supplemental Material Table S2 (http://dx.doi.org/10.1289/ehp.1104763) shows exposure by study area; there is a spatial exposure variation for most of the pollutants. Some chemical components pairs were highly correlated (Table 3). For example, ammonium ion had correlations of 0.74 and 0.73 with nitrate and sulfate, respectively, likely due to the common form of ammonium nitrate and ammonium sulfate. Other correlated pairs were calcium and zinc, nickel and vanadium, and nickel and zinc (correlations 0.63–0.64). Exposure to O_3_ negatively correlated with some PM_2.5_ chemical components (e.g., –0.68 with nickel). The highest and lowest correlations for any region are available in Supplemental Material, Table S3 (http://dx.doi.org/10.1289/ehp.1104763).

**Table 2:** Summary statistics of gestational pollutant exposures.

Pollutant	Mean ± SD	IQR
PM10 total mass (μg/m3)	22.34 ± 4.31	4.93
PM2.5 total mass (μg/m3)	13.41 ± 2.05	2.71
PM2.5 chemical components (μg/m3)			
Aluminum (Al)	0.019 ± 0.010	0.010
Ammonium ion (NH4+)	1.827 ± 0.437	0.50
Arsenic (As)	0.00116 ± 0.00056	0.0005
Cadmium (Cd)	0.00159 ± 0.00086	0.0013
Calcium (Ca)	0.046 ± 0.023	0.021
Chlorine (Cl)	0.037 ± 0.031	0.035
Elemental carbon (EC)	0.801 ± 0.324	0.335
Lead (Pb)	0.005 ± 0.003	0.0022
Mercury (Hg)	0.001 ± 0.001	0.0008
Nickel (Ni)	0.006 ± 0.006	0.0071
Nitrate (NO3–)	1.836 ± 0.705	0.90
OCM	3.593 ± 0.964	1.10
Silicon (Si)	0.07474 ± 0.03037	0.033
Sodium ion (Na+)	0.154 ± 0.095	0.076
Sulfate (SO42–)	4.148 ± 0.895	1.21
Titanium (Ti)	0.00417 ± 0.00176	0.0022
Vanadium (V)	0.00434 ± 0.00260	0.0043
Zinc (Zn)	0.019 ± 0.010	0.015
Gaseous pollutants (ppm)
CO	0.529 ± 0.194	0.214
NO2	0.021 ± 0.007	0.011
O3	0.023 ± 0.005	0.007
SO2	6.08 ± 2.52	3.16

**Table 3:** Pearson correlation of gestational pollutant exposures.

	PM2.5	Al	NH4+	As	Cd	Ca	Cl	EC	Pb	Hg	Ni	NO3–	OCM	Si	Na+	SO42–	Ti	V	Zn	CO	NO2	O3	SO2
PM10	0.44	0.18	0.36	0.15	0.29	0.15	–0.04	0.35	0.18	0.33	–0.02	0.07	0.36	0.16	–0.06	0.46	0.26	0.15	0.06	0.09	0.27	0.19	–0.10
PM2.5		0.12	0.82	0.38	0.32	0.25	0.12	0.44	0.35	0.30	0.20	0.49	0.56	0.38	–0.07	0.81	0.50	0.14	0.35	0.06	0.26	–0.12	0.39
Al			0.08	0.01	–0.09	0.27	0.05	0.28	0.06	–0.02	–0.15	–0.15	0.07	0.49	–0.16	0.12	0.14	0.02	0.07	–0.09	–0.03	0.22	–0.07
NH4+				0.40	0.28	0.30	0.23	0.36	0.36	0.31	0.15	0.74	0.47	0.24	–0.07	0.73	0.38	0.09	0.33	–0.01	0.25	–0.12	0.26
As					0.38	–0.02	0.28	0.11	0.78	0.38	–0.19	0.22	0.17	0.13	–0.16	0.40	0.06	–0.24	0.22	–0.30	–0.28	0.10	0.12
Cd						–0.05	–0.08	0.14	0.20	0.58	–0.09	0.20	0.18	0.14	–0.14	0.26	0.27	0.05	–0.02	0.00	–0.02	0.06	0.06
Ca							0.23	0.44	0.21	0.05	0.39	0.29	0.24	0.28	0.15	0.09	0.46	0.40	0.64	0.03	0.40	–0.28	0.39
Cl								0.26	0.25	–0.15	0.20	0.37	0.20	0.08	0.08	–0.08	–0.02	0.17	0.44	–0.05	0.15	–0.32	0.19
EC									0.25	0.14	0.49	0.27	0.53	0.15	0.01	0.16	0.35	0.50	0.59	0.37	0.65	–0.52	0.49
Pb										0.26	0.03	0.24	0.26	0.16	–0.01	0.28	0.22	–0.10	0.42	–0.16	–0.08	0.02	0.29
Hg											–0.11	0.18	0.12	0.00	–0.20	0.27	0.18	0.01	0.03	–0.11	–0.06	0.07	0.01
Ni												0.30	0.23	0.07	0.27	–0.06	0.25	0.64	0.63	0.34	0.72	–0.68	0.61
NO3–													0.32	0.06	0.11	0.16	0.22	0.23	0.34	0.12	0.41	–0.52	0.36
OCM														0.21	0.20	0.38	0.42	0.33	0.31	0.18	0.45	–0.09	0.25
Si															0.18	0.37	0.50	0.11	0.15	0.11	0.06	0.07	0.12
Na+																–0.08	0.24	0.29	0.22	0.15	0.29	–0.23	0.01
SO42–																	0.41	–0.09	0.12	–0.06	–0.03	0.20	0.09
Ti																		0.26	0.35	0.30	0.33	–0.08	0.24
V																			0.38	0.33	0.68	–0.57	0.26
Zn																				0.06	0.47	–0.59	0.58
CO																					0.55	–0.28	0.27
NO2																						–0.77	0.53
O3																							–0.61

Associations between confounder variables and LBW, which exclude pollutant exposures, were generally consistent with previous research indicating higher risks of LBW for female infants, first births, or infants with mothers who were African American, unmarried, or had started prenatal care after the first 3 months of pregnancy [see Supplemental Material, Table S4 (http://dx.doi.org/10.1289/ehp.1104763)]. Lower maternal education attainment was associated with LBW. A U-shaped relationship was observed for maternal age, with higher risk at low or high ages. There were no statistically significant differences in risk of LBW by birth year, but there were differences by region. For instance, relative risk is higher in urban areas (e.g., Manhattan, New York), than rural areas (e.g., New Hampshire).

Interquartile range (IQR) increases in PM_2.5_ chemical components of aluminum, calcium, elemental carbon, nickel, silicon, titanium, and zinc were significantly associated with LBW (Table 4). IQR increases in PM_10_, CO, NO_2_, and SO_2_ also were positively associated with LBW, whereas O_3_ showed a statistically significant negative association with LBW. When evaluated among first births only, the significant associations with chemical components remained except for silicon (*p* = 0.0504). Associations were no longer significant for PM_10_ and the gaseous pollutants, although the directions of the associations were unchanged (Table 4).

**Table 4:** Percent change in odds of LBW per IQR increment in pollutant for the gestational period (95% CI).

Pollutant	Original data	First births only
PM10 total mass (μg/m3)	3.2 (0.7, 5.8)**	4.0 (–0.2, 8.3)*
PM2.5 total mass (μg/m3)	2.2 (–0.2, 4.8)*	3.2 (–0.7, 7.3)
Aluminum (Al)	4.9 (3.4, 6.5)**	4.7 (2.1, 7.2)**
Ammonium ion (NH4+)	–0.4 (–2.5, 1.8)	–1.1 (–4.5, 2.4)
Arsenic (As)	–0.9 (–2.3, 0.6)	–0.7 (–3.0, 1.6)
Cadmium (Cd)	1.1 (–1.5, 3.7)	4.2 (0.1, 8.5)**
Calcium (Ca)	3.0 (1.6, 4.3)**	3.9 (1.7, 6.1)**
Chlorine (Cl)	–0.8 (–2.3, 0.8)	0.0 (–2.4, 2.5)
Elemental carbon (EC)	4.7 (3.2, 6.2)**	4.8 (2.5, 7.3)**
Lead (Pb)	0.0 (–1.3, 1.2)	–0.1 (–2.1, 1.9)
Mercury (Hg)	0.9 (–1.5, 3.4)	–2.7 (–6.5, 1.2)
Nickel (Ni)	5.7 (2.7, 8.8)**	6.5 (1.6, 11.5)**
Nitrate (NO3–)	–2.5 (–5.1, 0.2)*	–1.2 (–5.4, 3.2)
OCM	0.5 (–1.0, 2.1)	–0.2 (–2.6, 2.3)
Silicon (Si)	1.4 (0.0, 2.9)**	2.3 (0.0, 4.7)*
Sodium ion (Na+)	–0.9 (–2.0, 0.2)*	–0.6 (–2.3, 1.2)
Sulfate (SO42–)	–2.5 (–5.4, 0.5)	–2.4 (–7.1, 2.4)
Titanium (Ti)	5.0 (3.1, 7.0)**	5.5 (2.4, 8.7)**
Vanadium (V)	–1.7 (–4.9, 1.6)	–1.0 (–6.0, 4.4)
Zinc (Zn)	4.4 (1.7, 7.2)**	5.5 (1.2, 10.1)**
Gaseous pollutants (ppm)				
CO	3.3 (1.5, 5.1)**	0.8 (–2.0, 3.8)
NO2	4.7 (1.4, 8.1)**	1.1 (–3.9, 6.4)
O3	–6.3 (–11, –1.3)**	–5.5 (–13.1, 2.8)
SO2	3.1 (0.8, 5.5)**	2.5 (–1.0, 6.1)
Each logistic regression model was adjusted by maternal race, marital status, and tobacco and alcohol consumption during pregnancy, mother’s highest education, and mother’s age; infant sex; gestational length; the trimester prenatal care began; first in birth order; delivery method; average AT for each trimester; season of birth; year of birth; and regional indicators. IQR for each pollutant is listed in Table 2. *p = 0.05–0.1. **p < 0.05.				

We estimated effects by trimester for all pollutants that were significantly associated with LBW in single-pollutant models and report ranges of trimester-specific associations that were consistent across the main model and three sensitivity models (Table 5). Statistically significant associations were found for aluminum (all trimesters), calcium, nickel, silicon, and zinc (third trimester), elemental carbon and titanium (first trimester), and protective effect for O_3_ (first trimester). No consistent trimester results were found for other chemical components or gaseous pollutants (data not shown).

**Table 5:** Percent change in risk of LBW per IQR increment in pollutant for trimester exposure.

Pollutant	Trimester	Lowest effect to highest effect across multiple models
PM10 total mass	3rd	2.8 to 3.0
Aluminum	1st	1.5 to 2.6
	2nd	1.7 to 3.0
	3rd	1.6 to 2.6
Calcium	3rd	2.5 to 2.8
Elemental carbon	1st	3.1 to 4.3
Nickel	3rd	3.4 to 5.0
Silicon	3rd	1.3 to 1.4
Titanium	1st	2.1 to 3.5
Zinc	3rd	2.1 to 3.0
O3	1st	–5.0 to –4.7
Results are presented for pollutants and trimesters with consistent significant associations across the trimester models referenced in the methods section. Numbers are the range of effect in the alternative trimester models. No consistent trimester associations were observed for CO, NO2, and SO2. Each model was adjusted by maternal race, marital status, tobacco and alcohol consumption during pregnancy, highest education, and age; infant sex; gestational length; the trimester prenatal care began; first in birth order; delivery method; averaged AT for each trimester; season of birth; year of birth; and regional indicators.				

**Figure 1:**
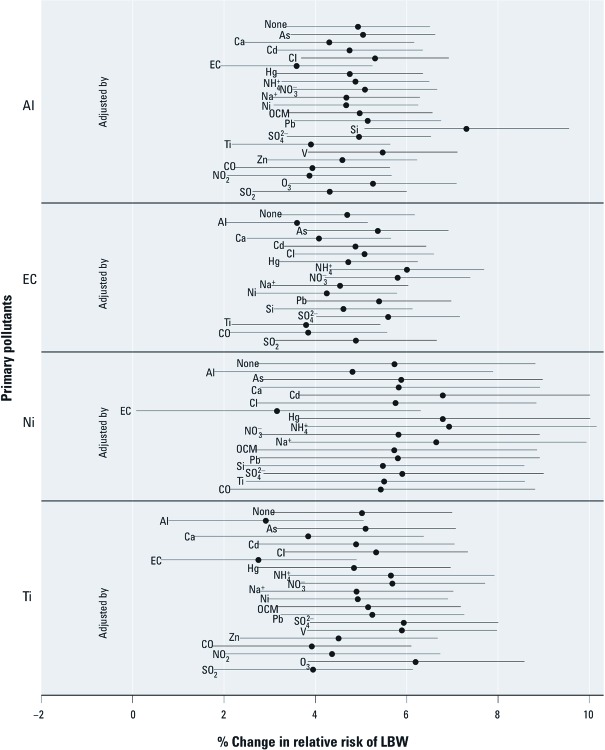
Percent change in relative risk of LBW per IQR increment in selected pollutants for gestational exposure with single (labeled as “None”) and two-pollutant (including the pollutant listed to the left of the estimates plus the pollutant indicated next to each estimate) logistic regression models. The point represents the central estimate and the horizontal line represents the 95% CI. See Table 4 for abbreviations.

Only associations between LBW and aluminum, elemental carbon, nickel, and titanium were robust to adjustment for all co-pollutants with correlation < 0.5 (Figure 1). Results for other pollutants (calcium, silicon, zinc, CO, NO_2_, O_3_, SO_2_, PM_10_) were generally robust, but were not statistically significant after adjustment for at least one co-pollutant [see Supplemental Material, Figure S1 (http://dx.doi.org/10.1289/ehp.1104763)].

For pollutants with consistent associations with LBW in two-pollutant models (PM_2.5_ aluminum elemental carbon, nickel, and titanium), we investigated whether associations differed by race or sex. The relative risk of LBW associated with an IQR increase in PM_2.5_ elemental carbon was 7.3% (95% CI: 4.9, 9.6%) lower among infants of African-American mothers compared with white mothers, and 3.2% (95% CI: 0.8, 5.6%) lower for females compared with males. The relative odds of LBW with an IQR increase in PM_2.5_ nickel were 10.2% (95% CI: 7.9, 12.4%) lower among infants of African-American mothers than for white mothers, and 4.6% (95% CI: 2.2, 7.1%) lower for females than for males. Associations between aluminum and titanium and LBW did not exhibit statistically significant differences by race or sex (data not shown).

## Discussion

To the best of our knowledge, this is the largest study to explore the association between PM_2.5_ chemical composition and pregnancy outcomes. Chemical components of aluminum, calcium, elemental carbon, nickel, silicon, titanium, and zinc were identified as potentially harmful, whereas statistically significant positive associations were not observed for ammonium ion, arsenic, cadmium, chlorine, lead, mercury, nitrate, organic carbon matter, sodium ion, sulfate, or vanadium. Of the components, results for aluminum, elemental carbon, nickel, and titanium were robust to co-pollutant adjustment. These chemical components likely result from different sources. Although all components have multiple sources, traffic emissions are the major source of PM_2.5_ elemental carbon, oil combustion is the major source of PM_2.5_ nickel, road dust is the major source of PM_2.5_ aluminum, and crustal material is a primary source of PM_2.5_ titanium (Bell et al. 2007a; Hains et al. 2007). Our results are consistent with our previous study conducted in Connecticut and Massachusetts, where PM_2.5_ aluminum, elemental carbon, and nickel were associated with LBW (Bell et al. 2010).

Previous studies have reported associations between chemical component exposures and a range of health outcomes. For example, PM_2.5_ elemental carbon was associated with hospitalization for childhood respiratory-related disease, and PM_2.5_ nickel was associated with cardiovascular-related hospitalization (Ito et al. 2011; Ostro et al. 2009). We identified associations between birth outcomes and multiple PM_2.5_ chemical components. As potential future work, researchers may apply source appointment or other methods to identify the origin of harmful pollutants (Lall et al. 2011), but source misclassification would be a potential concern given the size of our study region and heterogeneous distribution of PM_2.5_ chemical components and sources (Bell et al. 2011). Location-specific source apportionment analysis may be necessary for large study areas or when the distribution of PM_2.5_ sources varies within a study area.

For gaseous pollutants, LBW was associated with exposure to CO, NO_2_, and SO_2_. Our results also indicated a negative association between O_3_ and LBW. Some of these results (i.e., CO, NO_2_, SO_2_) are similar to those from previous studies (Darrow et al. 2011; Wu et al. 2011). However, none of the gaseous pollutants were significantly associated with LBW in first-birth-only analyses or based on two-pollutant models. This may indicate that previous pregnancy history is not fully taken into account in our model, or that gaseous pollutants are acting as surrogates for other pollutants. Other statistical approaches are needed to clarify potential effects of these exposures, such as longitudinal models or more sophisticated multipollutant models.

Associations between LBW and individual pollutants differed by trimester. Higher exposure of specific pollutants in the first trimester may relate to placenta development, whereas exposure in later stages may affect maternal vascular alteration, which causes the fetal growth retardation (Lin and Santolaya-Forgas 1999; Mannes et al. 2005). We found statistically significant associations with LBW for exposure during the first trimester to PM_2.5_ aluminum, elemental carbon, and titanium; for exposure in the second trimester for PM_2.5_ aluminum; and for exposure during the third trimester to PM_10_ and PM_2.5_ aluminum, calcium, nickel, silicon, and zinc. Some of these trimester results are consistent with our previous research in Connecticut and Massachusetts (Bell et al. 2010); however, other studies have reported associations with exposures during different trimesters. For instance, a study in California found that exposures to PM_10_ and PM_2.5_ in the first trimester were associated with LBW (Morello-Frosch et al. 2010), and in Spain exposure to NO_2_ in the first trimester was associated with LBW (Ballester et al. 2010). These inconsistencies might relate to differences in the study area or study design. Another potential reason is misclassification of the gestational exposure, because many studies, including the present study, determine gestational exposure based on the LMP and gestational length reported by birth certificate. LMP is likely reported as an approximate date rather than the actual LMP, resulting in a less accurate delivery date (Bell et al. 2007b). This approximation could lead to exposure misclassification that would have a larger effect on trimester-specific exposures than on average gestational exposures. Further studies are needed using actual birth date along with gestational week. Additional study is needed to better understand effects by trimester, which may inform understanding of high risk periods by exposing ambient air pollutants.

We observed that associations of LBW with PM_2.5_ elemental carbon and nickel were stronger among male infants than female infants and among infants of white mothers than infants of African-American mothers. These findings differ from a previous study that reported stronger associations between LBW and PM_2.5_ total mass among infants of African-American mothers than among those of white mothers (Bell et al. 2007b). This issue warrants further study to better understand susceptibilities.

The biological mechanisms that may contribute to effects of air pollution on birth outcomes are uncertain, and various hypotheses exist. For instance, NO_2_ exposure during pregnancy may limit placental vascular function and disturb fetal growth (Clifton et al. 2001). CO may react with oxygen on hemoglobin-binding sites, reducing oxygen delivery (Maisonet et al. 2004). Fetal growth may be retarded by direct toxic effects of air pollution, similar to effects of smoking (Ritz and Yu 1999). The mechanism of PM effects on birth outcomes could be related to the transfer of toxic components to the fetus from PM that has accumulated in the mother’s lungs (Ritz et al. 2007). PM has a complex chemical composition, and its chemical components may affect outcomes through different biological pathways. One possible explanation is that exposure to PM_2.5_ metal-related components, including aluminum and titanium, increases oxidative stress burdens leading to adverse health outcomes (Wei et al. 2009). There is a need for further studies to understand how individual PM_2.5_ chemical components and combinations of components affect the fetus.

Limitations of this study include the reliance on birth certificate data. Some previous works have described shortcomings regarding birth certificate variables, especially for tobacco and alcohol use, prenatal care, pregnancy complications, and labor (Dobie et al. 1998; Northam and Knapp 2006). In fact, our results showed unknown smoking status as a risk factor for LBW [see Supplemental Material, Table S4 (http://dx.doi.org/10.1289/ehp.1104763)], suggesting that those with unknown smoking status were more likely to have been smokers than nonsmokers, because maternal tobacco consumption affects LBW (Darrow et al. 2006; DiFranza et al. 2004; Horta et al. 1997; Parker and Woodruff 2008). On the other hand, several researchers investigated the reliability and validity of birth certificate data, and concluded that the data are adequate for adjustment purposes, though they warranted caution (Honein et al. 2001; Roohan et al. 2003). The reliability and validity of birth certificate data are not fully known; however, the key variables of interest for our study (i.e., birth weight, residence) are likely to be reliable and have some of the highest validity of any birth certificate variables (Northam and Knapp 2006; Shaw and Malcoe 1992). A further challenge is that levels of some chemical components, such as arsenic, might be below the minimum detection limit, which could lead to exposure misclassification. In our data, > 25% of arsenic measurements were zero, which may be attributable to levels that were below the detection limit. Another limitation is that we estimated exposures by residential county at birth, and were not able to incorporate actual address or prior residences if mothers moved during pregnancy. In addition, this approach does not address spatial heterogeneity of pollutants within a county, which may be particularly important for larger counties (Peng and Bell 2010). Exposure misclassification may occur for residents living far from monitors. In our data, the maximum distance from a monitor to the border of a county was 75.6 km (Essex County, NY). A recent study showed that correlations between levels of some PM_2.5_ chemical components were low for paired monitors that were < 10 km away (Bell et al. 2011). Our analysis omitted many births because many counties do not have PM_2.5_ chemical component monitors [see Supplemental Material, Table S1 (http://dx.doi.org/10.1289/ehp.1104763)]. Further, ambient monitors are warranted at more locations and with more frequent observations. Monitors in suburban and rural counties are particularly needed because monitors tend to be in urban counties, which may hinder study of the full range of population characteristics (Bravo and Bell 2011; Miranda et al. 2011). A larger study could also address potential differences in effects across types of locations, such as urban versus rural, because most of the counties in our data set were urban. In terms of residential mobility during pregnancy, our approach may not introduce substantial misclassification because recent studies found that most moving takes place within a short distance, though this is worthy of future studies (Bell and Belanger 2012; Madsen et al. 2010). Further limitations are that birth certificate data do not contain parental weight or genetic information. Several studies have reported that these factors are also linked to LBW (Freathy et al. 2010; Frederick et al. 2008).

## Conclusions

We found evidence of links between air pollution—including PM_2.5_ chemical components and gaseous pollutants—and LBW. We observed these associations even though most of our study region, except for a few large city areas, was in compliance with the National Ambient Air Quality Standards for PM_2.5_ and PM_10_, and all of the study region was in compliance with regulatory standards for CO, SO_2_, and NO_2_ (U.S. EPA 2009). Our results suggest that prenatal exposures to some PM_2.5_ chemical components may be more harmful than others, but current regulations are based exclusively on particle size and mass concentration. Our findings also suggest that even if two regions had identical levels of PM_2.5_ total mass, one might have levels of PM_2.5_ chemical components that result in higher risks of LBW. This is likely true for other health outcomes; our previous studies found that some specific chemical components are associated with hospital admission (Bell et al. 2009). Further scientific evidence on which components and sources of PM_2.5_ are most harmful would aid decision makers in developing policies intended to protect public health. Additional studies covering different regions, using more detailed birth data, and investigating other birth outcomes (such as preterm birth and small for gestational age) are needed to estimate the differential toxicity of various types of air pollutants, including PM_2.5_ chemical components, on birth outcomes.

## Supplemental Material

(356 KB) PDF
