# Supplementary material for: Airborne PM2.5 Chemical Components and Low Birth Weight in the Northeastern and Mid-Atlantic Regions of the United States
Source: Environ Health Perspect. 2012 Sep 20;120(12):1746–52. doi: 10.1289/ehp.1104763 (PMC3548298; doi:10.1289/ehp.1104763)

**Supplemental Material**

# Airborne $PM_{2.5}$ Chemical Components and Low Birth Weight in the Northeastern and Mid-Atlantic Regions of the United States

**Authors:** Keita Ebisu, Michelle L. Bell

## Supplemental Material Table of Contents

Supplemental Material, Table S1.......................................................................... *pg. S 2*
Supplemental Material, Table S2.......................................................................... *pg. S 5*
Supplemental Material, Table S3..........................................................................*pg. S 8*
Supplemental Material, Table S4..........................................................................*pg. S 9*
Supplemental Material, Figure S1........................................................................ *pg. S 11*

Supplemental Material, Table S1. Characteristics of births not included in analysis

| | Subjects living in counties without monitors but otherwise eligible | Subjects excluded based on criteria other than availability of pollutant data (including births in counties with and without monitors) |
|---|---|---|
| **Subject Number** | 4,868,521 | 1,022,094 |
| **Birth Weight (g) [Mean +/- Standard Deviation]** | 3421.0 +/- 474.8 | 2567.4 +/- 810.6 (missing for 11,171 observations) |
| **Low Birth Weight (< 2,500g)** | 120,212 (2.5%) | 423,328 (41.4%) |
| **Sex** | | |
| Male | 2,481,112 (51.0%) | 537,989 (52.6%) |
| Female | 2,387,409 (49.0%) | 484,105 (47.4%) |
| **Race** | | |
| Caucasian | 3,774,477 (77.5%) | 711,004 (69.6%) |
| African American | 787,846 (16.2%) | 253,616 (24.8%) |
| Other | 306,198 (6.3%) | 57,474 (5.6%) |
| **Maternal Age** | | |
| < 20 years | 376,303 (7.7%) | 90,989 (8.9%) |
| 20 to 24 | 985,526 (20.2%) | 200,446 (19.6%) |
| 25 to 29 | 1,255,300 (25.8%) | 239,539 (23.4%) |
| 30 to 34 | 1,357,389 (27.9%) | 270,508 (26.5%) |
| 35 to 39 | 733,206 (15.1%) | 171,923 (16.8%) |
| ≥ 40 | 160,797 (3.3%) | 48,689 (4.8%) |
| **Maternal Marital Status** | | |
| Married | 3,312,950 (68.1%) | 616,691 (60.3%) |
| Single | 1,555,571 (32.0%) | 405,403 (39.7%) |
| **Maternal Education** | | |
| Less than high school | 713,789 (14.7%) | 177,959 (17.4%) |
| High school | 1,383,804 (28.4%) | 300,892 (29.4%) |
| Some college | 1,064,182 (21.9%) | 218,191 (21.4%) |
| College | 1,653,608 (34.0%) | 307,419 (30.1%) |
| Unknown | 53,138 (1.1%) | 17,633 (1.7%) |
| **Maternal Alcohol Consumption During Pregnancy** | | |
| Yes | 33,528 (0.7%) | 7,635 (0.8%) |
| No | 3,695,037 (75.9%) | 727,308 (71.2%) |
| Unknown | 1,139,956 (23.4%) | 287,151 (28.1%) |
| **Maternal Tobacco Consumption During Pregnancy** | | |
| Yes | 488,132 (10.0%) | 119,121 (11.7%) |
| No | 4,361,384 (89.6%) | 895,083 (87.6%) |
| Unknown | 19,005 (0.4%) | 7,890 (0.8%) |

Supplemental Material, Table S1 (Continued).

| | Subjects living in counties without monitors but otherwise eligible | Subjects excluded based on criteria other than availability of pollutant data (including births in counties with and without monitors) |
|---|---|---|
| **Length of Gestation** | | |
| Less than 37 weeks | - | 690,835 (67.6%) |
| 37 to 38 | 1,380,171 (28.4%) | 83,812 (8.2%) |
| 39 to 40 | 2,571,607 (52.8%) | 27,388 (2.7%) |
| 41 to 42 | 785,231 (16.1%) | 6,270 (0.6%) |
| 43 to 44 | 131,512 (2.7%) | 1,745 (0.2%) |
| More than 44 | - | 66,840 (6.5%) |
| Missing | - | 145,204 (14.2%) |
| **Month Prenatal Care Begin** | | |
| First 3 months of pregnancy | 3,925,708 (80.6%) | 766,431 (75.0%) |
| 4th to 6th months of pregnancy | 622,946 (12.8%) | 150,417 (14.7%) |
| 7th month of pregnancy or later | 142,858 (2.9%) | 29,333 (2.9%) |
| No care | 34,360 (0.7%) | 23,706 (2.3%) |
| Unknown | 142,649 (2.9%) | 52,207 (5.1%) |
| **Birth Order** | | |
| First baby | 1,590,853 (32.7%) | 293,270 (28.7%) |
| Not first baby | 3,247,781 (66.7%) | 716,739 (70.1%) |
| Unknown | 29,887 (0.6%) | 12,085 (1.2%) |
| **Region** | | |
| Connecticut | 262,445 (5.4%) | 42,150 (4.1%) |
| Delaware | 36,740 (0.8%) | 14,238 (1.4%) |
| Massachusetts | 496,394 (10.2%) | 86,444 (8.5%) |
| Maryland and DC | 448,176 (9.2%) | 105,320 (10.3%) |
| New Hampshire | 74,873 (1.5%) | 14,444 (1.4%) |
| New Jersey | 638,518 (13.1%) | 138,247 (13.5%) |
| Manhattan area, New York | 920,307 (18.9%) | 212,027 (20.7%) |
| New York other than Manhattan area | 384,049 (7.9%) | 74,267 (7.3%) |
| Eastern PA | 408,817 (8.4%) | 107,474 (10.5%) |
| Western PA | 286,569 (5.9%) | 61,874 (6.1%) |
| Rhode Island | 55,937 (1.2%) | 14,500 (1.4%) |
| Virginia | 684,951 (14.1%) | 118,587 (11.6%) |
| Vermont | 37,144 (0.8%) | 5,963 (0.6%) |
| West Virginia | 133.601 (2.7%) | 26,559 (2.6%) |
| **Birth Year** | | |
| 2000 | 757,935 (15.6%) | 124,996 (12.2%) |
| 2001 | 722,177 (14.8%) | 123,682 (12.1%) |
| 2002 | 629,606 (12.9%) | 124,120 (12.1%) |
| 2003 | 561,124 (11.5%) | 126,839 (12.4%) |

Supplemental Material, Table S1 (Continued).

| | Subjects living in counties without monitors but otherwise eligible | Subjects excluded based on criteria other than availability of pollutant data (including births in counties with and without monitors) |
|---|---|---|
| 2004 | 574,729 (11.8%) | 128,407 (12.6%) |
| 2005 | 535,474 (11.0%) | 128,551 (12.6%) |
| 2006 | 541,745 (11.1%) | 132,485 (13.0%) |
| 2007 | 545,728 (11.2%) | 133,014 (13.0%) |
| **Birth Season** | | |
| Winter | 1,155,503 (23.7%) | 249,816 (24.4%) |
| Spring | 1,232,265 (25.3%) | 256,810 (25.1%) |
| Summer | 1,203,625 (24.7%) | 248,630 (24.3%) |
| Fall | 1,277,128 (26.2%) | 266,838 (26.1%) |

Supplemental Material, Table S2. Summary statistics of gestational pollutant exposures by each region

| | All | Connecticut | Delaware | Massachusetts | Maryland and DC | New Hampshire |
|---|---|---|---|---|---|---|
| Number of Infants | 1,207,800 | 33,246 | 40,412 | 51,674 | 111,187 | 26,314 |
| Number of Counties | 49 | 2 | 2 | 2 | 5 | 2 |
| $PM_{10}$ Total Mass (μg/m$^3$) | 22.3 (4.3) | 25.6 (5.9) | 23.1 (2.0) | 21.3 (2.3) | 19.7 (5.7) | 17.3 (2.0) |
| $PM_{2.5}$ Total Mass (μg/m$^3$) | 13.4 (2.1) | 12.6 (1.2) | 13.9 (1.5) | 12.1 (1.2) | 14.1 (1.4) | 9.3 (0.8) |
| $PM_{2.5}$ Chemical Components (μg/m$^3$) | | | | | | |
| Aluminum | 0.019 (0.01) | 0.017 (0.006) | 0.022 (0.014) | 0.015 (0.008) | 0.022 (0.009) | 0.013 (0.005) |
| Ammonium Ion | 1.8 (0.4) | 1.5 (0.2) | 2.1 (0.3) | 1.2 (0.1) | 1.8 (0.3) | 0.9 (0.1) |
| Arsenic | 0.0012 (0.0006) | 0.0009 (0.0002) | 0.0013 (0.0003) | 0.0008 (0.0002) | 0.0009 (0.0002) | 0.0009 (0.0002) |
| Cadmium | 0.0016 (0.0009) | 0.0020 (0.0010) | 0.0023 (0.0012) | 0.0015 (0.0008) | 0.0012 (0.0007) | 0.0010 (0.0002) |
| Calcium | 0.046 (0.023) | 0.030 (0.003) | 0.058 (0.039) | 0.032 (0.014) | 0.039 (0.008) | 0.032 (0.010) |
| Chlorine | 0.037 (0.031) | 0.021 (0.012) | 0.039 (0.026) | 0.050 (0.036) | 0.018 (0.014) | 0.048 (0.023) |
| Elemental Carbon | 0.801 (0.324) | 0.866 (0.1324) | 0.661 (0.165) | 0.654 (0.165) | 0.712 (0.144) | 0.491 (0.094) |
| Lead | 0.0049 (0.0027) | 0.0029 (0.0009) | 0.0038 (0.0011) | 0.0026 (0.0007) | 0.0037 (0.0011) | 0.0030 (0.0005) |
| Mercury | 0.0011 (0.0006) | 0.0011 (0.0004) | 0.0013 (0.0006) | 0.0008 (0.0004) | 0.0010 (0.0006) | 0.0006 (0.0002) |
| Nickel | 0.0061 (0.0064) | 0.0052 (0.0017) | 0.0035 (0.0012) | 0.0030 (0.0007) | 0.0012 (0.0008) | 0.0019 (0.0005) |
| Nitrate | 1.8 (0.7) | 1.4 (0.2) | 2.2 (0.5) | 1.1 (0.2) | 1.5 (0.5) | 0.7 (0.2) |
| Organic Carbon Matter | 3.6 (1.0) | 3.3 (0.4) | 3.6 (1.1) | 3.5 (0.9) | 3.5 (1.0) | 3.5 (0.9) |
| Silicon | 0.075 (0.030) | 0.053 (0.010) | 0.102 (0.051) | 0.057 (0.028) | 0.086 (0.028) | 0.058 (0.015) |
| Sodium Ion | 0.154 (0.095) | 0.133 (0.028) | 0.160 (0.088) | 0.163 (0.086) | 0.117 (0.083) | 0.161 (0.087) |
| Sulfate | 4.1 (0.90) | 3.6 (0.6) | 4.6 (0.7) | 3.0 (0.3) | 4.7 (0.8) | 2.7 (0.3) |
| Titanium | 0.0042 (0.0018) | 0.0030 (0.0012) | 0.0058 (0.0029) | 0.0035 (0.0018) | 0.0053 (0.0023) | 0.0029 (0.0007) |
| Vanadium | 0.0043 (0.0026) | 0.0088 (0.0040) | 0.006 (0.0022) | 0.0036 (0.001) | 0.0025 (0.0006) | 0.0041 (0.0012) |
| Zinc | 0.019 (0.010) | 0.011 (0.002) | 0.015 (0.009) | 0.015 (0.010) | 0.014 (0.004) | 0.014 (0.004) |
| CO (ppm) | 0.529 (0.194) | 0.567 (0.131) | 0.429 (0.070) | 0.393 (0.112) | 0.711 (0.323) | 0.523 (0.102) |
| $NO_2$ (ppm) | 0.021 (0.007) | 0.021 (0.002) | 0.019 (0.002) | 0.019 (0.003) | 0.019 (0.004) | 0.010 (0.002) |
| $O_3$ (ppm) | 0.023 (0.005) | N.A. | 0.025 (0.004) | 0.023 (0.004) | 0.024 (0.004) | 0.032 (0.000) |
| $SO_2$ (ppb) | 6.1 (2.5) | 4.0 (0.8) | 4.7 (1.2) | 4.2 (1.4) | 5.4 (1.6) | 4.0 (1.3) |

Supplemental Material, Table S2 (continued).

| | New Jersey | Manhattan, New York | New York Other than Manhattan | Eastern Pennsylvania | Western Pennsylvania |
|---|---|---|---|---|---|
| Number of Infants | 146,508 | 344,901 | 82,194 | 203,428 | 100,355 |
| Number of Counties | 4 | 3 | 4 | 10 | 6 |
| $PM_{10}$ Total Mass (μg/m$^3$) | 27.5 (3.6) | 21.5 (2.8) | 7.7 (1.3) | 23.6 (4.1) | 22.6 (4.0) |
| $PM_{2.5}$ Total Mass (μg/m$^3$) | 12.7 (1.9) | 13.8 (1.5) | 11.5 (1.9) | 14.8 (1.8) | 14.7 (1.9) |
| $PM_{2.5}$ Chemical Components (μg/m$^3$) | | | | | |
| Aluminum | 0.022 (0.015) | 0.017 (0.006) | 0.013 (0.006) | 0.019 (0.008) | 0.023 (0.010) |
| Ammonium Ion | 1.7 (0.3) | 1.9 (0.2) | 1.7 (0.3) | 2.2 (0.4) | 1.9 (0.4) |
| Arsenic | 0.0010 (0.0002) | 0.0009 (0.0002) | 0.0012 (0.0005) | 0.0014 (0.0004) | 0.0022 (0.0008) |
| Cadmium | 0.0020 (0.0008) | 0.0013 (0.0005) | 0.0009 (0.0003) | 0.0020 (0.0009) | 0.0021 (0.0009) |
| Calcium | 0.035 (0.017) | 0.064 (0.021) | 0.051 (0.033) | 0.040 (0.011) | 0.037 (0.010) |
| Chlorine | 0.025 (0.018) | 0.048 (0.029) | 0.031 (0.021) | 0.037 (0.032) | 0.047 (0.051) |
| Elemental Carbon | 0.864 (0.520) | 1.013 (0.272) | 0.483 (0.160) | 0.731 (0.146) | 0.854 (0.350) |
| Lead | 0.0043 (0.0015) | 0.0047 (0.0013) | 0.0065 (0.0038) | 0.0051 (0.0016) | 0.0088 (0.0040) |
| Mercury | 0.0013 (0.0006) | 0.0009 (0.0004) | 0.0008 (0.0004) | 0.0014 (0.0007) | 0.0015 (0.0008) |
| Nickel | 0.0037 (0.0014) | 0.0148 (0.0055) | 0.0005 (0.0002) | 0.0032 (0.0017) | 0.0017 (0.0011) |
| Nitrate | 1.7 (0.5) | 2.1 (0.4) | 1.8 (0.6) | 2.5 (0.9) | 1.4 (0.4) |
| Organic Carbon Matter | 3.5 (1.3) | 3.8 (0.8) | 2.9 (0.7) | 3.8 (0.9) | 3.4 (0.8) |
| Silicon | 0.071 (0.034) | 0.074 (0.017) | 0.064 (0.024) | 0.078 (0.041) | 0.085 (0.027) |
| Sodium Ion | 0.149 (0.094) | 0.200 (0.112) | 0.172 (0.077) | 0.128 (0.073) | 0.092 (0.036) |
| Sulfate | 4.0 (0.7) | 4.1 (0.5) | 3.6 (0.7) | 4.5 (0.8) | 5.0 (1.0) |
| Titanium | 0.0043 (0.0019) | 0.0046 (0.0013) | 0.0032 (0.0013) | 0.0037 (0.0015) | 0.0040 (0.0014) |
| Vanadium | 0.0043 (0.0024) | 0.0066 (0.0011) | 0.0013 (0.0005) | 0.0036 (0.0018) | 0.0013 (0.0005) |
| Zinc | 0.014 (0.007) | 0.029 (0.006) | 0.014 (0.006) | 0.016 (0.005) | 0.024 (0.011) |
| CO (ppm) | 0.599 (0.206) | 0.602 (0.112) | 0.457 (0.049) | 0.438 (0.155) | 0.308 (0.126) |
| $NO_2$ (ppm) | 0.020 (0.008) | 0.028 (0.004) | 0.016 (0.002) | 0.020 (0.005) | 0.014 (0.003) |
| $O_3$ (ppm) | 0.028 (0.004) | 0.018 (0.003) | 0.027 (0.004) | 0.022 (0.003) | 0.024 (0.003) |
| $SO_2$ (ppb) | 4.2 (1.0) | 8.1 (2.7) | 5.3 (1.7) | 5.2 (1.4) | 7.4 (2.1) |

Supplemental Material, Table S2 (continued).

| | Rhode Island | Virginia | Vermont | West Virginia |
|---|---|---|---|---|
| Number of Infants | 31,108 | 20,399 | 8,653 | 7,421 |
| Number of Counties | 1 | 5 | 1 | 2 |
| $PM_{10}$ Total Mass ($\mu g/m^3$) | 19.4 (2.6) | 26.5 (4.5) | 15.0 (3.3) | 22.9 (2.1) |
| $PM_{2.5}$ Total Mass ($\mu g/m^3$) | 10.9 (1.0) | 13.6 (1.3) | 9.0 (1.0) | 15.3 (1.8) |
| $PM_{2.5}$ Chemical Components ($\mu g/m^3$) | | | | |
| Aluminum | 0.013 (0.004) | 0.019 (0.009) | 0.013 (0.004) | 0.029 (0.015) |
| Ammonium Ion | 1.1 (0.1) | 1.7 (0.2) | 1.0 (0.1) | 1.6 (0.2) |
| Arsenic | 0.0013 (0.0010) | 0.0012 (0.0004) | 0.0005 (0.0001) | 0.0013 (0.0003) |
| Cadmium | 0.0008 (0.0004) | 0.0020 (0.0008) | 0.0021 (0.0008) | 0.0018 (0.0008) |
| Calcium | 0.026 (0.005) | 0.024 (0.005) | 0.024 (0.004) | 0.031 (0.006) |
| Chlorine | 0.039 (0.022) | 0.009 (0.004) | 0.007 (0.004) | 0.014 (0.010) |
| Elemental Carbon | 0.542 (0.067) | 0.536 (0.084) | 0.321 (0.044) | 0.587 (0.066) |
| Lead | 0.0055 (0.0065) | 0.0035 (0.0015) | 0.0020 (0.0004) | 0.0035 (0.0013) |
| Mercury | 0.0007 (0.0004) | 0.0014 (0.0005) | 0.0012 (0.0004) | 0.0015 (0.0006) |
| Nickel | 0.0040 (0.0015) | 0.0016 (0.0006) | 0.0009 (0.0002) | 0.0012 (0.0004) |
| Nitrate | 1.1 (0.2) | 1.1 (0.3) | 0.8 (0.2) | 0.7 (0.2) |
| Organic Carbon Matter | 3.1 (0.7) | 4.7 (0.8) | 2.7 (0.6) | 3.0 (0.4) |
| Silicon | 0.065 (0.020) | 0.069 (0.020) | 0.057 (0.014) | 0.094 (0.023) |
| Sodium Ion | 0.154 (0.026) | 0.143 (0.103) | 0.123 (0.081) | 0.066 (0.016) |
| Sulfate | 3.1 (0.4) | 4.9 (0.8) | 2.2 (0.4) | 5.2 (0.9) |
| Titanium | 0.0031 (0.0008) | 0.0034 (0.0014) | 0.0024 (0.0009) | 0.0032 (0.0008) |
| Vanadium | 0.0063 (0.0023) | 0.0016 (0.0006) | 0.0011 (0.0002) | 0.0014 (0.0009) |
| Zinc | 0.010 (0.002) | 0.011 (0.009) | 0.006 (0.001) | 0.010 (0.001) |
| CO (ppm) | 0.553 (0.093) | 0.403 (0.048) | 0.490 (0.110) | N.A. |
| $NO_2$ (ppm) | 0.017 (0.003) | 0.019 (0.002) | 0.013 (0.002) | N.A. |
| $O_3$ (ppm) | N.A. | 0.031 (0.002) | 0.034 (0.002) | 0.028 (0.002) |
| $SO_2$ (ppb) | 5.0 (1.3) | 3.9 (0.5) | 2.5 (0.1) | 9.7 (0.8) |

Supplemental Material, Table S3. Minimum and maximum of Pearson correlations for any region.

*Note*: The minimum correlation for any region is provided in the top row. The maximum correlation for any region is provided in the bottom row.

| | $PM_{2.5}$ | Al | $NH_4^+$ | As | Cd | Ca | Cl | EC | Pb | Hg | Ni | $NO_3^-$ | OCM | Si | $Na^+$ | $SO_4^=$ | Ti | V | Zn | CO | $NO_2$ | $O_3$ | $SO_2$ |
|---|---|---|---|---|---|---|---|---|---|---|---|---|---|---|---|---|---|---|---|---|---|---|
| **$PM_{10}$** | -0.10 | -0.61 | -0.19 | -0.64 | -0.54 | -0.58 | -0.60 | -0.30 | -0.56 | -0.35 | -0.70 | -0.68 | -0.77 | -0.28 | -0.67 | 0.08 | -0.59 | -0.59 | -0.67 | -0.53 | -0.67 | 0.12 | -0.61 |
| | 0.99 | 0.40 | 0.86 | 0.80 | 0.67 | 0.78 | 0.68 | 0.89 | 0.81 | 0.68 | 0.62 | 0.54 | 0.87 | 0.67 | 0.23 | 0.96 | 0.80 | 0.66 | 0.79 | 0.87 | 0.88 | 0.77 | 0.63 |
| **$PM_{2.5}$** | | -0.36 | 0.34 | -0.18 | -0.07 | -0.12 | -0.36 | -0.37 | -0.25 | -0.18 | -0.34 | -0.60 | 0.35 | 0.13 | -0.32 | 0.60 | 0.19 | -0.13 | -0.58 | -0.43 | -0.48 | -0.73 | -0.50 |
| | | 0.54 | 0.92 | 0.76 | 0.66 | 0.68 | 0.47 | 0.65 | 0.75 | 0.61 | 0.63 | 0.58 | 0.85 | 0.58 | 0.44 | 0.93 | 0.79 | 0.71 | 0.63 | 0.70 | 0.68 | 0.68 | 0.77 |
| **Al** | | | -0.21 | -0.57 | -0.77 | -0.19 | -0.40 | -0.43 | -0.54 | -0.74 | -0.41 | -0.49 | -0.31 | -0.24 | -0.49 | -0.23 | -0.66 | -0.49 | -0.41 | -0.84 | -0.56 | 0.01 | -0.64 |
| | | | 0.44 | 0.48 | 0.02 | 0.80 | 0.62 | 0.59 | 0.67 | 0.08 | 0.45 | 0.22 | 0.72 | 0.86 | 0.53 | 0.30 | 0.61 | 0.52 | 0.72 | 0.37 | 0.35 | 0.74 | 0.31 |
| **$NH_4^+$** | | | | -0.18 | -0.02 | -0.26 | -0.33 | -0.38 | -0.07 | -0.14 | -0.38 | -0.09 | -0.03 | -0.15 | -0.50 | 0.51 | 0.03 | -0.35 | -0.27 | -0.47 | -0.70 | -0.72 | -0.42 |
| | | | | 0.72 | 0.50 | 0.76 | 0.61 | 0.70 | 0.64 | 0.33 | 0.81 | 0.79 | 0.88 | 0.66 | 0.32 | 0.90 | 0.72 | 0.69 | 0.65 | 0.62 | 0.70 | 0.56 | 0.70 |
| **As** | | | | | 0.08 | -0.32 | -0.28 | -0.78 | 0.13 | -0.09 | -0.47 | -0.08 | -0.40 | -0.65 | -0.28 | -0.01 | -0.22 | -0.21 | -0.08 | -0.64 | -0.40 | -0.93 | -0.09 |
| | | | | | 0.75 | 0.62 | 0.70 | 0.82 | 0.98 | 0.74 | 0.63 | 0.70 | 0.76 | 0.57 | 0.44 | 0.57 | 0.62 | 0.68 | 0.86 | 0.88 | 0.86 | 0.26 | 0.54 |
| **Cd** | | | | | | -0.26 | -0.65 | -0.72 | -0.13 | -0.16 | -0.61 | -0.15 | -0.25 | -0.48 | -0.36 | -0.04 | -0.12 | -0.24 | -0.35 | -0.41 | -0.23 | -0.80 | -0.09 |
| | | | | | | 0.60 | 0.30 | 0.50 | 0.72 | 0.78 | 0.73 | 0.49 | 0.51 | 0.58 | 0.24 | 0.30 | 0.79 | 0.72 | 0.44 | 0.80 | 0.81 | 0.03 | 0.74 |
| **Ca** | | | | | | | -0.42 | -0.40 | -0.35 | -0.44 | -0.65 | -0.21 | -0.42 | -0.36 | -0.49 | -0.22 | -0.05 | -0.38 | -0.17 | -0.46 | -0.35 | -0.70 | -0.41 |
| | | | | | | | 0.77 | 0.85 | 0.69 | 0.49 | 0.74 | 0.59 | 0.80 | 0.96 | 0.71 | 0.69 | 0.94 | 0.80 | 0.77 | 0.57 | 0.74 | 0.66 | 0.64 |
| **Cl** | | | | | | | | -0.30 | -0.35 | -0.64 | -0.30 | -0.16 | -0.34 | -0.37 | -0.43 | -0.41 | -0.55 | -0.25 | -0.15 | -0.35 | -0.21 | -0.39 | -0.37 |
| | | | | | | | | 0.78 | 0.72 | 0.23 | 0.71 | 0.75 | 0.60 | 0.67 | 0.40 | 0.27 | 0.52 | 0.64 | 0.87 | 0.68 | 0.71 | 0.76 | 0.80 |
| **EC** | | | | | | | | | -0.68 | -0.65 | -0.25 | -0.46 | -0.35 | -0.53 | -0.76 | -0.36 | -0.56 | -0.47 | -0.22 | -0.79 | -0.73 | -0.55 | -0.27 |
| | | | | | | | | | 0.81 | 0.41 | 0.83 | 0.71 | 0.86 | 0.76 | 0.82 | 0.50 | 0.75 | 0.80 | 0.81 | 0.84 | 0.93 | 0.91 | 0.71 |
| **Pb** | | | | | | | | | | -0.31 | -0.65 | -0.05 | -0.14 | -0.48 | -0.26 | 0.01 | -0.09 | -0.20 | 0.02 | -0.59 | -0.25 | -0.52 | -0.34 |
| | | | | | | | | | | 0.75 | 0.72 | 0.62 | 0.71 | 0.76 | 0.51 | 0.47 | 0.73 | 0.85 | 0.91 | 0.74 | 0.71 | 0.28 | 0.67 |
| **Hg** | | | | | | | | | | | -0.29 | -0.25 | -0.52 | -0.63 | -0.46 | -0.04 | -0.42 | -0.21 | -0.51 | -0.37 | -0.48 | -0.83 | -0.27 |
| | | | | | | | | | | | 0.54 | 0.45 | 0.27 | 0.43 | 0.23 | 0.37 | 0.41 | 0.55 | 0.30 | 0.65 | 0.61 | 0.65 | 0.57 |
| **Ni** | | | | | | | | | | | | -0.23 | 0.02 | -0.37 | -0.81 | -0.23 | -0.41 | -0.02 | -0.45 | -0.82 | -0.24 | -0.67 | -0.23 |
| | | | | | | | | | | | | 0.80 | 0.69 | 0.49 | 0.55 | 0.62 | 0.67 | 0.99 | 0.61 | 0.80 | 0.87 | 0.98 | 0.65 |
| **$NO_3$** | | | | | | | | | | | | | -0.17 | -0.60 | -0.39 | -0.46 | -0.16 | -0.24 | -0.10 | -0.31 | -0.21 | -0.78 | 0.09 |
| | | | | | | | | | | | | | 0.67 | 0.46 | 0.69 | 0.20 | 0.51 | 0.76 | 0.81 | 0.91 | 0.94 | 0.28 | 0.91 |
| **OCM** | | | | | | | | | | | | | | -0.09 | -0.29 | 0.25 | -0.09 | -0.06 | -0.47 | -0.62 | -0.15 | -0.23 | -0.40 |
| | | | | | | | | | | | | | | 0.83 | 0.68 | 0.72 | 0.83 | 0.71 | 0.69 | 0.60 | 0.84 | 0.52 | 0.66 |
| **Si** | | | | | | | | | | | | | | | -0.32 | 0.00 | 0.07 | -0.36 | -0.55 | -0.62 | -0.16 | -0.13 | -0.61 |
| | | | | | | | | | | | | | | | 0.74 | 0.57 | 0.88 | 0.58 | 0.79 | 0.65 | 0.62 | 0.69 | 0.71 |
| **$Na^+$** | | | | | | | | | | | | | | | | -0.29 | -0.22 | -0.78 | -0.66 | -0.69 | -0.67 | -0.49 | -0.32 |
| | | | | | | | | | | | | | | | | 0.30 | 0.76 | 0.56 | 0.64 | 0.88 | 0.72 | 0.21 | 0.85 |
| **$SO_4^=$** | | | | | | | | | | | | | | | | | 0.23 | -0.07 | -0.49 | -0.29 | -0.86 | -0.91 | -0.43 |
| | | | | | | | | | | | | | | | | | 0.67 | 0.61 | 0.41 | 0.31 | 0.47 | 0.73 | 0.44 |
| **Ti** | | | | | | | | | | | | | | | | | | -0.28 | -0.47 | -0.66 | -0.37 | -0.18 | -0.59 |
| | | | | | | | | | | | | | | | | | | 0.71 | 0.61 | 0.68 | 0.83 | 0.39 | 0.70 |
| **V** | | | | | | | | | | | | | | | | | | | -0.30 | -0.84 | -0.58 | -0.86 | -0.30 |
| | | | | | | | | | | | | | | | | | | | 0.75 | 0.76 | 0.81 | 0.52 | 0.85 |
| **Zn** | | | | | | | | | | | | | | | | | | | | -0.51 | -0.30 | -0.65 | -0.14 |
| | | | | | | | | | | | | | | | | | | | | 0.72 | 0.78 | 0.27 | 0.84 |
| **CO** | | | | | | | | | | | | | | | | | | | | | -0.43 | -0.65 | -0.35 |
| | | | | | | | | | | | | | | | | | | | | | 0.92 | 0.13 | 0.87 |
| **$NO_2$** | | | | | | | | | | | | | | | | | | | | | | -0.72 | -0.16 |
| | | | | | | | | | | | | | | | | | | | | | | 0.29 | 0.89 |
| **$O_3$** | | | | | | | | | | | | | | | | | | | | | | | -0.76 |
| | | | | | | | | | | | | | | | | | | | | | | | 0.66 |

Supplemental Material, Table S4. Percent change in relative risk of low birth weight from logistic regression model without pollution variables (95% confidence interval)

| Category | Risk of Low Birth Weight (%) |
|---|---|
| **Sex** | |
| Male | -35.2 (-36.6, -33.7)** |
| Female | Reference |
| **Race** | |
| African American | 67.0 (62.8, 71.4)** |
| Other | 79.2 (72.6, 86.0)** |
| Caucasian | Reference |
| **Maternal Age** | |
| < 20 years | 6.0 (1.2, 11.1)** |
| 20 to 24 | 1.9 (-1.7, 5.6) |
| 25 to 29 | 0.1 (-3.1, 3.4) |
| 35 to 39 | 7.4 (3.3, 11.6)** |
| ≥ 40 | 38.9 (30.7, 47.5)** |
| 30 to 34 | Reference |
| **Maternal Marital Status** | |
| Unmarried | 33.4 (29.7, 37.2)** |
| Married | Reference |
| **Maternal Education** | |
| Less than high school | 9.8 (6.5, 13.2)** |
| Some College | -11.7 (-14.5, -8.9)** |
| College | -23.7 (-26.3, -21.0)** |
| Unknown | 5.6 (-4.7, 17.1) |
| High school | Reference |
| **Maternal Alcohol Consumption During Pregnancy** | |
| Yes | 39.6 (20.7, 61.5)** |
| Unknown | -3.0 (-7.3, 1.5) |
| No | Reference |
| **Maternal Tobacco Consumption During Pregnancy** | |
| Yes | 125.4 (117.9, 133.3)** |
| Unknown | 54.9 (37.2, 74.7)** |
| No | Reference |
| **Length of Gestation** | |
| 37 to 38 Weeks | 261.6 (253, 270.5)** |
| 41 to 42 | -15.4 (-18.8, -11.9)** |
| 43 to 44 | 30.0 (21.0, 39.6)** |
| 39 to 40 | Reference |
| **Month Prenatal Care Begin** | |
| 4th to 6th months of pregnancy | 18.0 (14.6, 21.6)** |
| 7th month of pregnancy or later | 20.5 (14.4, 27.0)** |

Supplemental Material, Table S4 (Continued).

| Category | Risk of Low Birth Weight (%) |
|---|---|
| No care | 150.2 (129.1, 173.4)** |
| Unknown | 36.8 (27.8, 46.4)** |
| First 3 months of pregnancy | Reference |
| **Birth Order** | |
| After first baby | -35.4 (-37.0, -33.8)** |
| Unknown | -16.8 (-26.9, -5.3)** |
| First Baby | Reference |
| **Delivery Method** | |
| Vaginal | -20.8 (-23.1, -18.3)** |
| Unknown | -18.8 (-22.6, -14.8)** |
| C-Section | Reference |
| **Region** | |
| Delaware | -4.9 (-13.4, 4.4) |
| Massachusetts | -6.3 (-14.2, 2.3) |
| Maryland and DC | -5.3 (-12.5, 2.5) |
| New Hampshire | -21.3 (-30.2, -11.2)** |
| New Jersey | -6.7 (-13.7, 0.8)* |
| Manhattan area, New York | 10.4 (2.6, 18.7)** |
| New York other than Manhattan area | -20.4 (-27.2, -12.9)** |
| Eastern PA | -9.5 (-17.3, -1.0)** |
| Western PA | -15.5 (-22.3, -8.1)** |
| Rhode Island | -2.3 (-11.5, 7.9) |
| Virginia | 10.4 (-0.4, 22.5)* |
| Vermont | -11.8 (-25.9, 4.9) |
| West Virginia | -6.0 (-18.8, 8.7) |
| Connecticut | Reference |
| **Birth Year** | |
| 2000 | 7.6 (-5.3, 22.4) |
| 2001 | 1.9 (-6.4, 10.9) |
| 2002 | -2.6 (-9.2, 4.4) |
| 2003 | -4.1 (-9.7, 1.9) |
| 2004 | -2.3 (-7.9, 3.7) |
| 2005 | -1.4 (-6.0, 3.3) |
| 2006 | -0.9 (-5.3, 3.8) |
| 2007 | Reference |
| **Birth Season** | |
| Winter | 3.0 (-3.3, 9.7) |
| Spring | 2.7 (-2.2, 7.9) |
| Fall | -6.4 (-10.9, -1.6)** |
| Summer | Reference |

*Note:* * p-value 0.05-0.1, ** p-value <0.05.

Supplemental Material, Figure S1. Percentage change in relative risk of low birth weight per interquartile increment in selected pollutants for gestational exposure with single and two pollutant logistic models. This figure shows effects for pollutants that were not depicted in Figure 1. The point is the central estimate and the vertical line represents the 95% confidence interval. The black circle represents significant associations ($p<0.05$). The white diamond represents non-significant associations ($p\geq0.05$).

a) Results for CO, and $NO_2$.

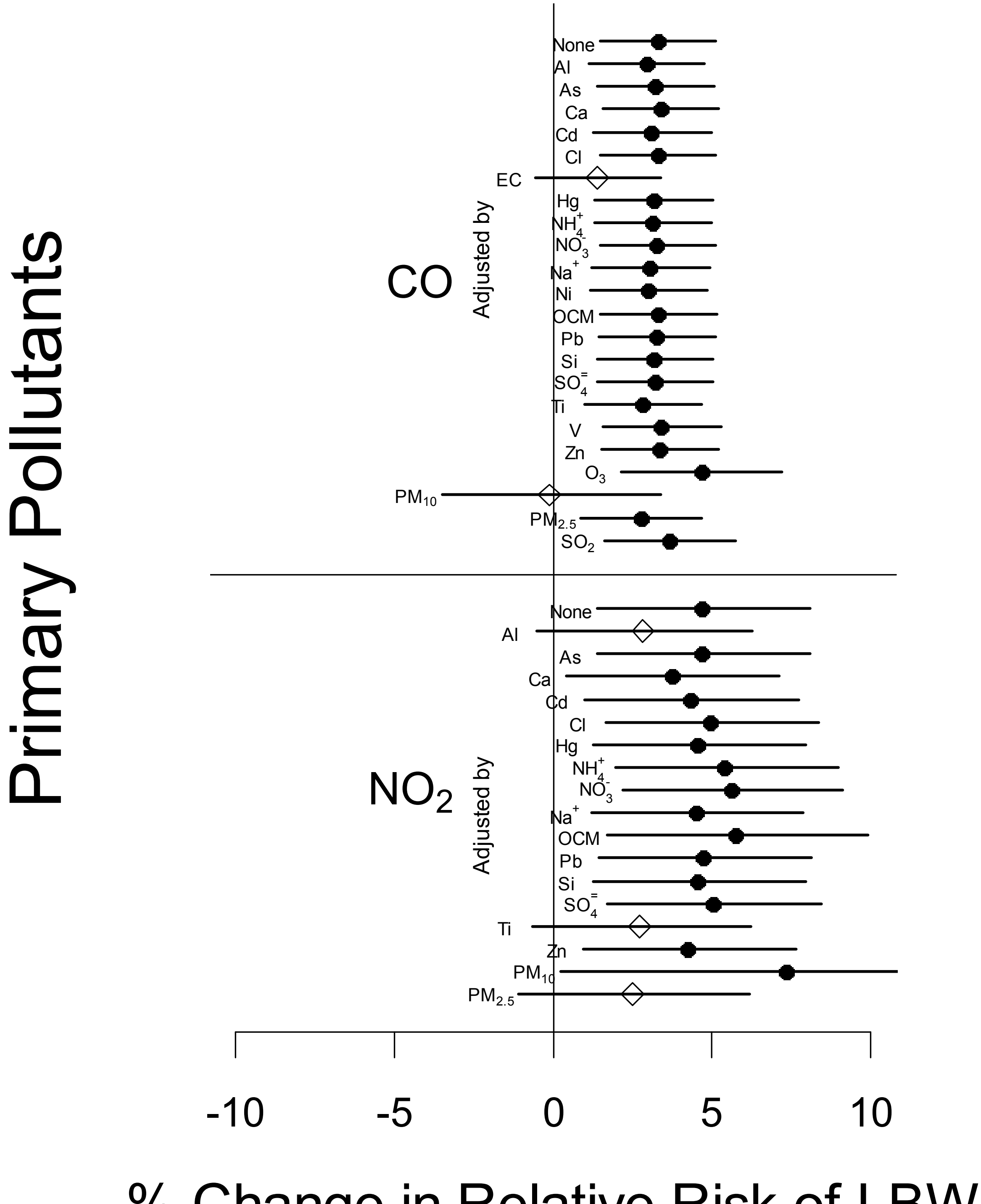

Supplemental Material, Figure S1 b) Results for $O_3$, $NO_2$, and $PM_{10}$.

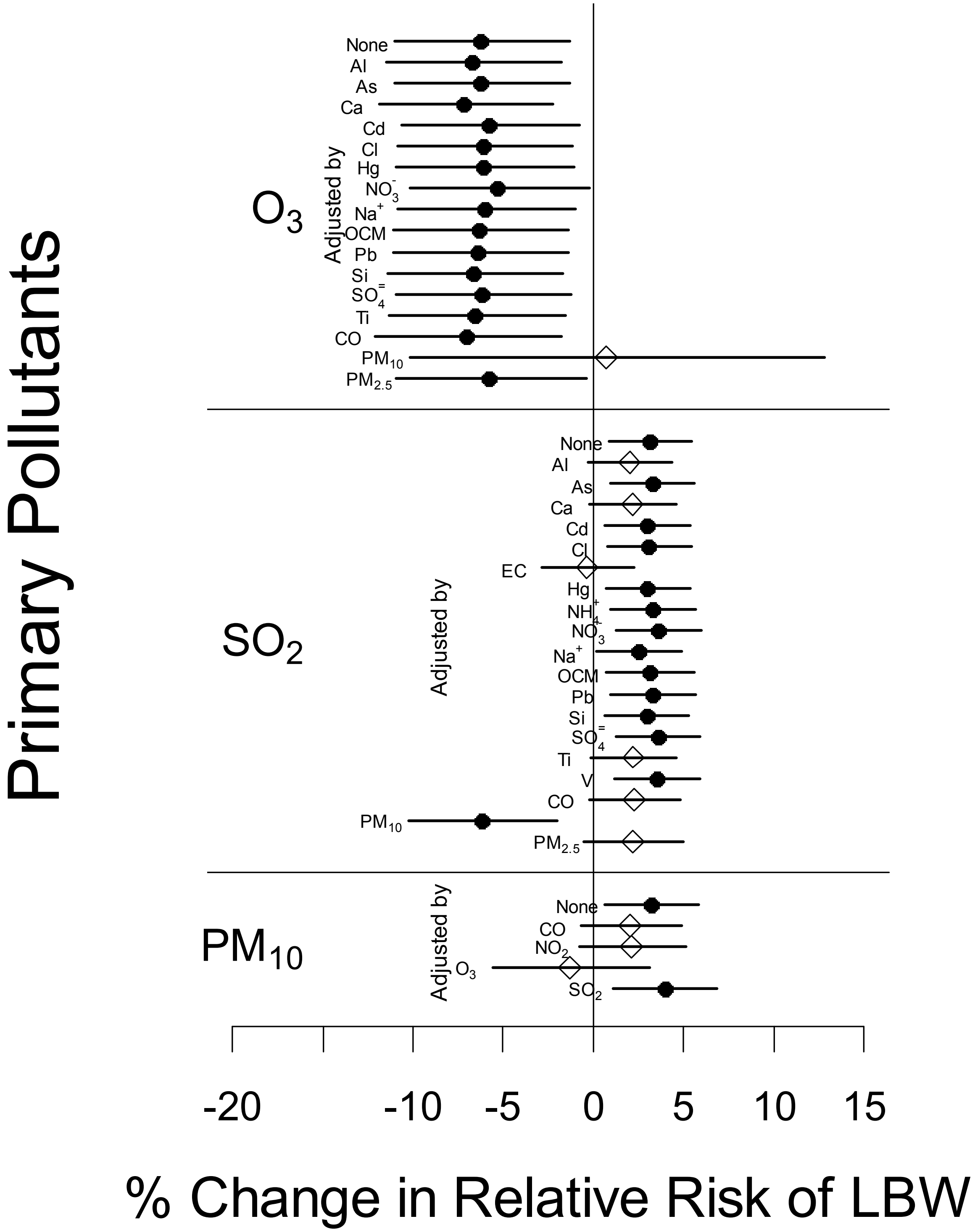

Supplemental Material, Figure S1 c) Results for Calcium, Silicon, and Zinc.

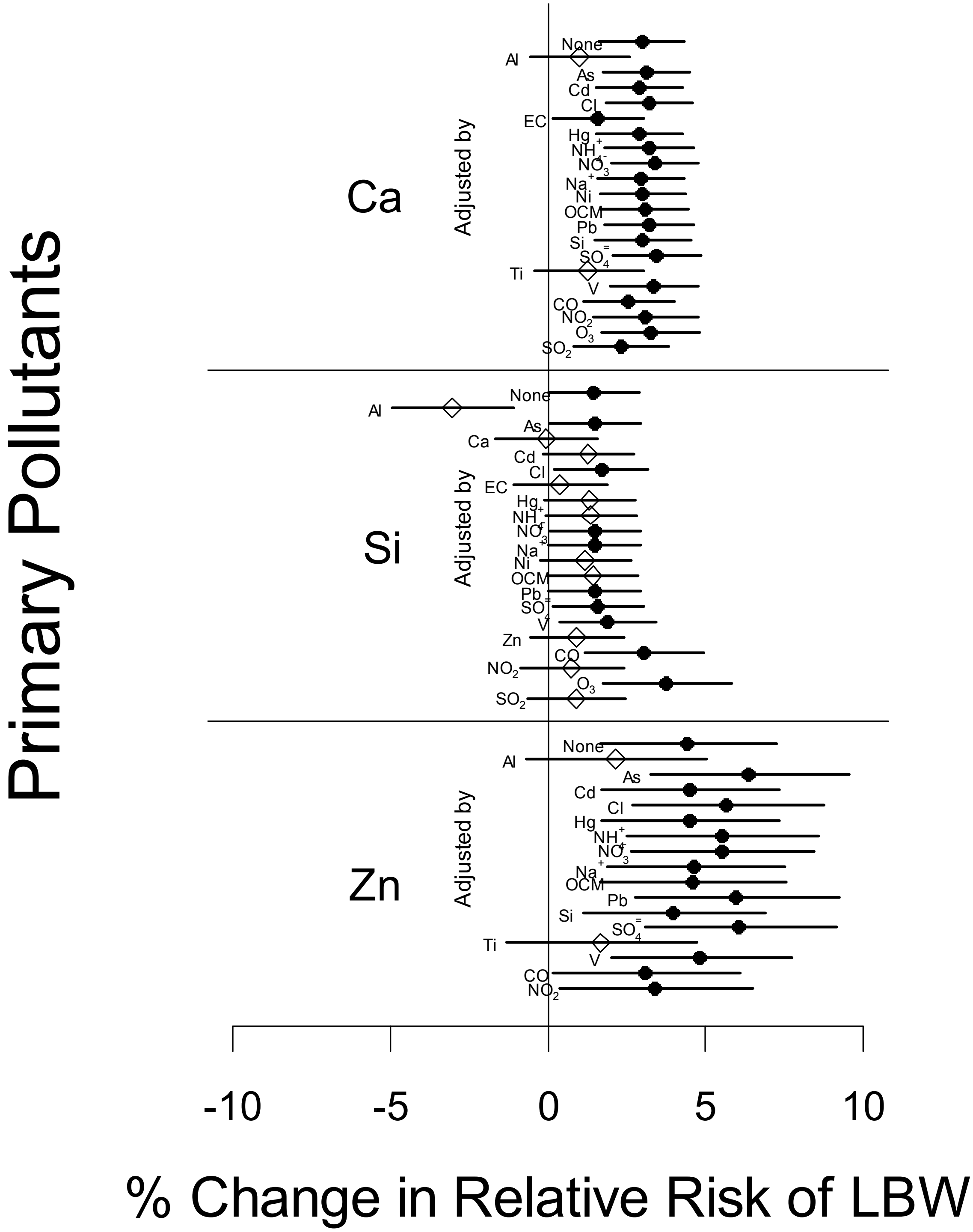

Supplement: (356 KB) PDF [file ehp.1104763.s001.pdf]
